# New Advances of Preimplantation and Prenatal Genetic Screening and Noninvasive Testing as a Potential Predictor of Health Status of Babies

**DOI:** 10.1155/2014/306505

**Published:** 2014-03-24

**Authors:** Tanya Milachich

**Affiliations:** SAGBAL Dr. Shterev, IVF Unit, Hristo Blagoev 25-31, 1330 Sofia, Bulgaria

## Abstract

The current morphologically based selection of human embryos for transfer cannot detect chromosome aneuploidies. So far, only biopsy techniques have been able to screen for chromosomal aneuploidies in the in vitro fertilization (IVF) embryos. Preimplantation genetic diagnosis (PGD) or screening (PGS) involves the biopsy of oocyte polar bodies or embryonic cells and has become a routine clinical procedure in many IVF clinics worldwide, including recent development of comprehensive chromosome screening of all 23 pairs of chromosomes by microarrays for aneuploidy screening. The routine preimplantation and prenatal genetic diagnosis (PND) require testing in an aggressive manner. These procedures may be invasive to the growing embryo and fetus and potentially could compromise the clinical outcome. Therefore the aim of this review is to summarize not only the new knowledge on preimplantation and prenatal genetic diagnosis in humans, but also on the development of potential noninvasive embryo and fetal testing that might play an important role in the future.

## 1. Introduction

A quarter of century has already passed since the first application of preimplantation genetic diagnosis (PGD) by Handyside in 1990 [1]. In the beginning, this method was applied for monogenic diseases and sex-linked disorders. Later, the major indications expanded for detection of chromosomal abnormalities and presence of translocations in either partner. The recent development of comprehensive chromosome screening of all 23 pairs of chromosomes by microarrays or the so-called preimplantation genetic screening (PGS) for aneuploidies and translocation in human embryos was achieved [2]. In the last decade, the PGD list was expanded for other purposes such as cancer predisposition disorders, rhesus incompatibility, mitochondrial disorders, and human leukocyte antigen typing [3–8]. Nowadays, PGD could be offered for any disorder for which molecular testing can be performed. The point of this technique is the removal of cells from the preimplantation embryos in the IVF program, genetic testing of these cells, and replacement (or freezing) of normal embryos into the uterus. In spite of the great advantage of PGD and its benefit for couples with genetic disorders (birth of a healthy baby or prevention of repeated spontaneous abortion), the embryo biopsy is an aggressive method, which may disturb the embryo. New approaches are being developed for indirect evaluation of the genetic status of human embryos in the IVF programs. The aim of this review is to summarize the recent knowledge on preimplantation (PGD) and prenatal genetic diagnosis (PND) and the potential use of noninvasive testing of embryos and fetuses in the future.

## 2. Preimplantation Genetic Diagnosis 

**Figure 1:**
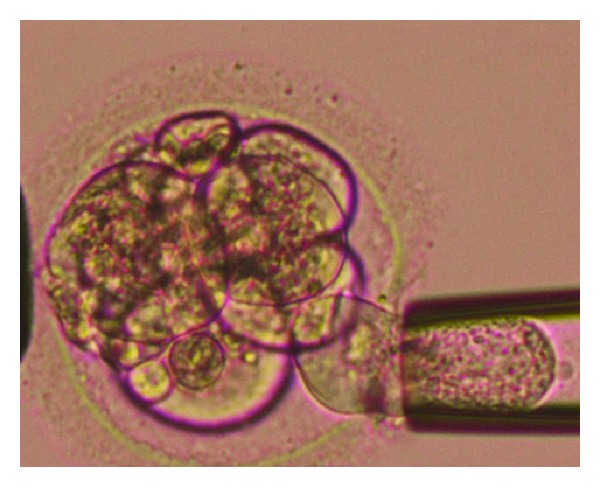
Preimplantation embryo biopsy in the in vitro fertilization program. Aspiration of a blastomere into the biopsy pipette.

PGD or preimplantation genetic screening (PGS) is performed at three different stages of the embryo development: (1) oocyte polar body biopsy [9] before and after fertilization, (2) blastomere biopsy [10] at cleavage stage (Figure 1), and (3) trophectoderm (TE) tissue biopsy at blastocyst stage [11]. There are certain pitfalls related to the genetic diagnosis of single cells such as amplification failure, preferential amplification, allele dropout (ADO), and contamination with extraneous DNA [12, 13]. Polar body or blastomere cells are more prone to these problems since they contain a limited amount of material available for genetic analysis. However, trophectoderm tissue biopsy at the blastocyst stage has the advantage of removing more cells (5–10), which potentially reduces the occurrence of these risks [11]. In addition to the technical advantages, blastocyst stage biopsy has the advantage of selecting developmentally more competent embryos for diagnosis that could improve pregnancy rates while at the same time it decreases the cost of the PGD study [14].

## 3. Oocyte Polar Body Biopsy

Biopsies of the first and second polar bodies have been performed for three decades [16], but nowadays only several countries use this technique routinely. The first polar body biopsy is applicable for couples with ethical concerns as preconception genetic diagnostic tool [17]. Another stage for biopsy is the period before syngamy (and after an ICSI procedure). The laws in several countries (e.g., Austria, Switzerland, and Germany) establish this procedure because it prohibits the genetic testing of cells derived from cleavage preimplantation embryo. Worldwide there are only few PGD laboratories where the genetic testing of the first and second polar bodies is still routinely used (e.g., RGI, Chicago, USA). Today this method is not a common practice. This method has more disadvantages than advantages such as lack of information for aneuploidies of paternal and mitotic origin; need of analysis of a huge amount of polar bodies and therefore unnecessary work and kits for diagnostic (some of the oocytes will not be fertilized and some of the zygotes will not reach the blastocyst stage); being highly expensive; chance for aneuploidy compensation (2–4%) [18]; high risk of aneuploidy (32.5%) [19]. Some advantages of this “early biopsy” are the diagnostics of oocytes themselves and female infertility, lack of mosaicism, and the minimal risk of affecting the embryo during the biopsy. However, there are still some perspectives, which could be used in reproductive medicine. The polar body genetic analysis might be an interesting approach—the aim is to select the oocytes after in vitro maturation procedure, which are appropriate for in vitro fertilization, and to improve the outcome of oocyte in vitro maturation in the clinical practice.

## 4. Preimplantation Embryo Biopsy

This is a biopsy for the later stages—the cleavage stage or the blastocyst stage embryo [11, 20]. However, day 3 embryo biopsy still possesses a high risk of mosaicism: from 55% to 73% [21–23]. In general, blastomere biopsy has limitations because of the fact that up to 60% of embryos at cleavage stage of development exhibit mosaicism, where at least one cell has a different ploidy from other cells in the embryo [24, 25]. Additionally, many cleavage stage embryos diagnosed as aneuploid with blastomere biopsy will “self-correct” by blastocyst stage, which, from a clinical stand point, may decrease the chances of a live birth by prematurely labeling an embryo as abnormal [26–30]. Even though, blastomere biopsies often successfully predict ploidy of the fetus, limitations such as mosaicism and self-correction complicate the issuing of a correct diagnosis, even when using highly accurate PGS technologies.

Mosaicism occurs also in blastocysts, but apparently at lower levels than in cleavage stage embryos. In a study of Johnson et al. [25] the rate of mosaicism between inner cell mass (ICM) and trophectoderm (TE), as well as between TE fractions, was only 3.9%. In addition, it was evident that the aneuploidy rate is significantly lower (38.8%) in blastocysts than in embryos at earlier stages (51%) [20]. Cleavage stage PGD/PGS could have negative impact on clinical outcome due to the embryo biopsy procedure whereas day 5 diagnosis (and freezing the biopsied embryos) allows the biopsy of cells that are not involved in the formation of the embryo rather than cells that may be committed to forming the ICM [11]. In addition to all these facts, the optimized uterine environment in the next cycle and the possibility of a single embryo transfer (SET) are in favor of day 5 embryo biopsy and genetic analysis. According to different studies this strategy may culminate in a pregnancy rate per transfer of 63% to 70.5% [31, 32].

## 5. Correlation between Genetic and Indirect Methods of Embryo Selection: Noninvasive Preimplantation Genetic Testing without Embryo Biopsy in the Future?

Is it possible for noninvasive preimplantation diagnosis to exist in the future as an unique tool? There is a tendency for noninvasive screening and searching correlations between different quality parameters of gametes, zygotes, embryos (vacuoles in sperm heads, spindle position in mature oocytes, cleavage intervals of zygote, and embryo developmental dynamics) and aneuploidy rates in human gametes and embryos. Different methods have been suggested for this approach.

### 5.1. Selection of Sperm for Fertilization

The intracytoplasmic morphologically selected sperm injection (IMSI) enables the selection of sperm for fertilization and improves poor embryo development in couples with poor semen quality. A randomized study of the team of Virant-Klun [33] showed that the IMSI procedure improves embryo development along with the laboratory and clinical outcomes of sperm microinjection in the same infertile couples with male infertility and poor embryo development over the previous ICSI attempts. Some studies have already confirmed that there is an increased aneuploidy rate in spermatozoa with large vacuoles in their heads [34]. The analysis of sperm, performed after morphological selection by high-magnification microscopy, indeed showed a significantly better mitochondrial function, chromatin status, and euploidy rate than observed in unselected cells. Moreover, a recent study showed that chromosomal architecture might be disturbed in spermatozoa with large vacuoles in their heads [35]. Therefore, it could be speculated that the selection of good-quality sperm could decrease the aneuploidy rates in the resulting embryos. As the technique seems noneffective for any unselected patients, relevant indications for the use of IMSI need to be defined. For patients with severe male factor evidence suggests higher clinical pregnancy and lower miscarriage rates [36]. In addition, it is known that the presence of all 23 pairs of chromosomes is a prerequisite for normal implantation and healthy fetal development in humans. The improved outcome of in vitro fertilization using IMSI was also observed in patients with teratozoospermia due to improved development and quality of embryos [37].

### 5.2. Blastocoele Fluid and DNA Extraction without Biopsy

**Figure 2:**
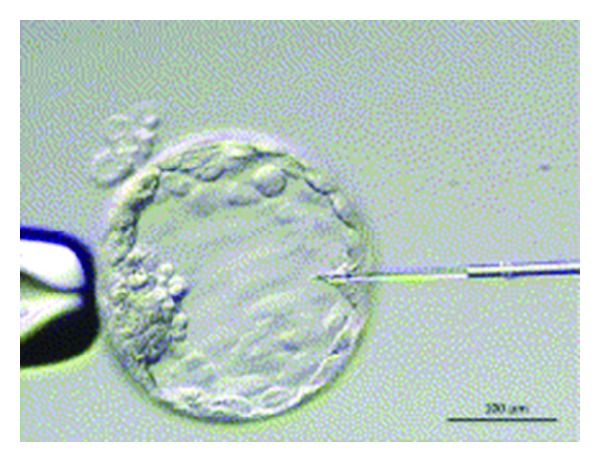
Aspiration of the blastocoel fluid using the ICSI pipette (source: [15]).

The analysis of the fluid from the blastocyst cavity (blastocoele) is an interesting approach. Using the real-time PCR, the study showed [15] that genomic DNA was present in about 90% of blastocoele fluid samples harvested during the vitrification procedure and this fluid could be obtained with ICSI pipette from blastocoel (Figure 2) avoiding any cell biopsy of the embryo. This method for blastocyst micropuncture and aspiration of blastocoele fluid has been described previously [38]. Briefly, the expanded day 5 blastocysts were removed from the culture medium and were transferred to a new droplet of blastocyst medium under paraffin oil. The blastocysts were immobilized with a holding pipette and another finely pulled, oil-filled pipette was introduced through the mural trophectoderm to avoid damaging the inner cell mass. Then the blastocoele fluid was aspirated gently until the blastocyst fully collapsed around the pipette.

The aim of this study was to determine the embryo gender directly from the blastocoele fluid without performing biopsy of embryonic cells. For this purpose the amplification of the multicopy genes* TSPY1* (on the Y chromosome) and* TBC1D3* (on chromosome 17) was done. This study opens up the possibility of screening embryos from couples carrying an X-linked disorder to identify male embryos at high risk of disease as well as detect several aneuploidies. However, further studies have to be done in order to validate this approach and to confirm that the accuracy is sufficient for diagnostic purposes [15].

The advantages of performing PGD without embryo biopsy are obvious, but this approach must be considered with caution before any potential clinical application. The group of Cohen [39] has some realistic concerns about this study. These are related to the DNA sample and the doubt that it does not represent the whole embryo since the embryo-free culture media also contain DNA fractions. In addition, it was suggested that this DNA has also been released from abnormal or degenerated cells and therefore could not be as representative as the one released from the intact ones. Moreover, the procedure may be called noninvasive but some damage may occur during the manipulation process and may affect the viability of the blastocyst. Many questions and many doubts arose, but in spite of them, the study of Palini and coauthors is interesting and fascinating and in a provocative manner opens new possibilities for diagnosis of genetic abnormalities in preimplantation embryos by avoiding any cell biopsy during the procedure [39].

### 5.3. Proteomics, Proteins in Spent Culture Media, and Noninvasive Testing of Embryos

This failure of embryo implantation from ART is due to both the absence of developmentally competent euploid embryos in an IVF cohort and our inability to select the competent embryo(s). In human, the incorrect number of chromosomes (aneuploidy) is extremely common in human oocytes and increases significantly with advanced maternal age [40, 41]. The embryos generated from these aneuploid gametes have little potential and reduced chance for a viable pregnancy [42]. New recent developments strategies in proteomic technologies and mass spectrometry (MS) have discovered differentially secreted proteins that could lead to noninvasive viability screening, including chromosomal constitution among preimplantation embryos [43]. A recent study of the team of Katz-Jaffe has found a potential biomarker for noninvasive aneuploidy screening called lipocalin-1. The researchers identified this protein in the secretome of human blastocysts in in vitro conditions. An important question could arise: may the protein secretome of human blastocysts be relative to comprehensive chromosome constitution in a noninvasive manner? The method is based on the analysis of proteins of the spent culture media, secreted by a single embryo (secretome). The difference between the euploid and aneuploid blastocyst in a unique secretome signature was evaluated. The preliminary results have been promising and revealed protein differences that appeared to correlate with chromosome constitution [43]. The protein secretome profiles from individual morphologically similar good-quality blastocysts allowed discrimination between euploid and aneuploid status. In this study, a novel set of nine differentially expressed biomarkers (soluble tumor necrosis factor (TNF), interleukin-10 (IL-10), macrophage-stimulating protein-*α* (MSP-*α*), stem cell factor (SCF), chemokine (C-X-C motif) ligand 13 (CXCL13), TNF-related apoptosis inducing ligand receptor 3 (TRAILR3), macrophage inflammatory protein-1*β* (MIP-1*β*), GM-CSF, and lipocalin-1) was identified with statistical significance and was reproducible in all of the analyzed spent culture media samples [44]. The protein profile of the euploid blastocyst secretome was notably different from the protein profile of the aneuploid blastocyst secretome. These biomarkers characteristically classified chromosome aneuploidy in the cohort of blastocysts available for transfer. The most significant suggestion in this study is that the altered expression levels of lipocalin-1 are related to aneuploidy and not to failed implantation, revealing their potential as a candidate marker for noninvasive aneuploidy screening. The development of this noninvasive technique for determining the euploidy and the competence for development of human embryos by analyzing the spent culture medium could be a powerful tool for embryo selection in ART, but it needs to be researched further.

### 5.4. Embryo Time-Lapse Monitoring and Aneuploidy

The application of time-lapse imaging of the embryos could be used as a predictor for good implantation and lower aneuploidy rate among the transferable embryos. The widely discussed study of Meseguer et al. [45] reported that morphokinetics of development could be used for prediction of embryo implantation and also could be associated with aneuploidy incidence. The time-lapse observation is an opportunity for optimizing embryo selection based on morphological grading as well as providing novel kinetic parameters, which may further improve accurate selection of viable embryos [46]. A detailed retrospective analysis of time-lapse microscopy results showed that several parameters of developmental dynamics were significantly correlated with subsequent implantation (e.g., time of first and subsequent cleavages as well as the time between cleavages). The most predictive parameters were (1) time of division to 5 cells, t5 (48.8–56.6 h after ICSI); (2) time between division to 3 cells and subsequent division to 4 cells, s2 (≤0.76 h); and (3) duration of the second cycle of the cell division, that is, time between division to 2 cells and division to 3 cells, cc2 (≤11.9 h).

The embryo aneuploidy, a major cause of IVF failure, has been correlated with specific embryonic morphokinetic variables previously used for the development of an aneuploidy risk classification model. The study of Campbell et al. [47] evaluates the effectiveness and potential impact of this model for unselected IVF patients without embryo biopsy and preimplantation genetic screening (PGS). Embryo outcomes, implantation, fetal heart beat (FHB), and live birth (LB)   of 88 transferred blastocysts were compared according to calculated aneuploidy risk classes (low, medium, and high). A significant difference was seen for FHB (*P* < 0.0001) and LB (*P* < 0.01) rates between embryos classified as low and medium risk. Within the low-risk class, relative increases of 74% and 56%, compared to rates for all blastocysts, were observed for FHB and LB, respectively. This study demonstrated the clinical relevance of the aneuploidy risk classification model and introduced a novel, noninvasive method of embryo selection in order to achieve higher implantation and live birth rates without PGS. By using such unique, noninvasive, and specifically designed embryo selection models, we can now make more informed choices in order to select the most viable embryo to transfer, with the lowest risk of aneuploidy. As a result of this study, the selection of an embryo, classified as low risk, has improved the relative chance of a live birth by 56% over conventional embryo selection.

## 6. Noninvasive Prenatal Diagnosis and Testing for Pregnant Women

Not only PGD and PGS, but also the noninvasive prenatal diagnosis (NIPD) and noninvasive prenatal testing (NIPT) will offer some new options in prenatal diagnosis for carriers of single gene disorders and chromosomal constitution in fetuses. This will involve fertile patients who reject PGD, patients after PGD for result confirmation, those who reject amniocentesis (AC) or chorionic villus sampling (CVS), patients with previous loss of pregnancy because of the listed procedures, and so forth. These carriers or patients at high risk for chromosomal or monogenic disorder are target groups for the health professionals working in the area of prenatal care.

The cell-free DNA from the fetus has been found in the plasma of pregnant women, and this has been used successfully for noninvasive determination of the fetal gender and fetal RhD genotype in RhD negative women [48–50]. The basis of these tests is the detection of fetal-specific DNA sequences in maternal plasma [51]. The same approach of searching for fetal-specific nucleic acids, such as DNA methylation and mRNA markers in maternal plasma, has been proposed for noninvasive detection of fetal aneuploidies [52, 53] instead of performing invasive sampling of fetal genetic material through the AC or CVS. As source for testing is the circulating in maternal blood 4–6% cell-free fetal DNA/RNA fraction in the 1st trimester of the pregnancy [54].

The noninvasive prenatal diagnosis (NIPD) for single-gene disorders has attracted less interest because it represents a much smaller market opportunity and in the majority of cases has to be provided on disease-specific basis. The methods and workflows are labour-intensive and not easily scalable. Nonetheless, there is a significant need of NIPD of single-gene disorders, and the continuing advances in technology and data analysis should facilitate the expansion of the variety of the disorders where NIPD can be provided. Various methods and platform technologies, as well as technical challenges, were applied to a wider range of genetic disorders. A recent report showed that these tests were mainly performed for haemophilia [55], beta-thalassaemia [56], and sickle cell anemia [57].

The other test is noninvasive prenatal testing (NIPT), which could be performed before the invasive testing (AC, CVS) for pregnant women who are considered having high risk of trisomy 21. According to Bianchi, this methodology has already been highly applicable for chromosome 21 [58]. There are many findings that besides chromosome 21 and sex chromosome aneuploidies, other chromosomes could also be analyzed (i.e., chromosomes 18 and 13). Nevertheless, the measurements of the proportion of DNA molecules from chromosomes 18 and 13 were far less precise [59]. In the future, further research is required to develop protocols in order to improve the precision for measuring the amount of DNA molecules from chromosomes 18 and 13 [54]. The recent study shows that the routine screening for trisomies: chromosomes 21, 18, and 13 by cell-free DNA (cfDNA) testing at 10 weeks of gestation is feasible and has lower false-positive rates (FPR) than combined testing does, but abnormal results require confirmation by CVS [60]. Time will show if the accuracy of NIPT is as high as the karyotyping after invasive procedure and if the invasive methods can be replaced by noninvasive genetic screening for pregnant women.

Since the ART and reproductive genetics are overlapping fields, necessity for collaboration between the genetic and ART centers has arisen.

## 7. Minimizing the Genetic Risk for Future ART Generations

Epimutation is also a hot topic, since many PGD laboratories already provide diagnosis for some syndromes and many recent articles search for correlation between ART and some imprinting disorders. Therefore, the field of epigenetic inheritance seems to be a quite interesting area, especially because ART can induce epigenetic variation that might be transmitted to the next generation [61].

The Angelman syndrome is a serious neurodevelopmental disorder [62] although there are no estimates of its absolute risk after ART would be small (1 in 3000). Therefore, it seems unlikely that this would result in many couples requesting ART to decline treatment. Epimutations causing Beckwith-Wiedemann syndrome (BWS) are more frequent than those causing Angelman syndrome but, not in comparison to the risk of serious complications such as exomphalos and embryonal tumours, BWS is usually compatible with normal living.

In order to provide prospective parents with accurate risk information, there is a pressing need to define the absolute risk of imprinting disorders after ART by prospectively following a cohort of ART children. It is acknowledged that many couples will still choose trying for pregnancy despite the known and unknown risks for the child [63].

Suboptimal conditions during oocyte and embryo development may also lead to persistent changes in the epigenome influencing diseases susceptibility later in life. In order to minimize the risk it is clear that the prolongation in vitro culturing to blastocyst stage should be very well optimized. The oocytes with big smooth endoplasmic reticulum (SER) aggregation might be followed by increased frequency of imprinting disorders. Therefore, their use for fertility treatment must be limited [64]. It is also not known how the embryo biopsy affects the embryo quality. However, apparently the highest risk for rare imprinting disorders in children born following ART remains multiple pregnancy and particularly higher-order multiple pregnancies [65].

Today a successful pregnancy is mainly defined by the outcome at birth; however, the consideration for the consequences of ART conditions for later life remains. The fetuses' adaptations to under nutrition are associated with changes in the concentrations of fetal and placental hormones. Maternal reproductive health is a reflection of events over generations. It is multifactorial, environmentally sensitive and involves genes undergoing reprogramming during the critical period of gametogenesis. It is now widely accepted that the adverse preconceptional and intrauterine environment is associated with epigenetic malprogramming of the fetal metabolism and predisposition to chronic, in particular metabolic disorders, later in life—or the so-called “Barker hypothesis” [66, 67], regardless if the child is born following assisted or natural conception.

There are also some new forthcoming horizons of the meaning of miRNA, siRNA, and piRNA that may play an important role in many biological processes [11], including differentiation of male reproductive cells, and they all may have control over the gene expression and need to be elucidated further.

## 8. Conclusion

It can be concluded that the preimplantation and prenatal genetic diagnosis and screening are of enormous value for providing healthy baby to couples with genetic disorders or for preventing the repeated spontaneous miscarriages. Nevertheless, there are some concerns about the aggression of the embryo biopsy by itself and potential epigenetic disturbance; therefore, there are some new noninvasive approaches for evaluation of the genetic status of human embryos and fetuses by a nondirect manner. Some of these approaches are interesting and seem to be quite promising, but further research is needed to elucidate if some of them could replace the existing procedures in the future or can only have additive value in diagnosis.
